# Computational Metabolomics Reveals the Potential Mechanism of Matrine Mediated Metabolic Network Against Hepatocellular Carcinoma

**DOI:** 10.3389/fcell.2022.859236

**Published:** 2022-07-22

**Authors:** Kexin Wang, Xiangmin Ye, Chuanhui Yin, Qing Ren, Yupeng Chen, Xuemei Qin, Chuanzhi Duan, Aiping Lu, Li Gao, Daogang Guan

**Affiliations:** ^1^ National Key Clinical Specialty/Engineering Technology Research Center of Education Ministry of China, Guangdong Provincial Key Laboratory on Brain Function Repair and Regeneration, Neurosurgery Institute, Department of Neurosurgery, Zhujiang Hospital, Southern Medical University, Guangzhou, China; ^2^ Institute of Integrated Bioinformedicine and Translational Science, Hong Kong Baptist University, Hong Kong SAR, China; ^3^ Department of Biochemistry and Molecular Biology, School of Basic Medical Sciences, Southern Medical University, Guangzhou, China; ^4^ School of Chinese Medicine, Li Ka Shing Faculty of Medicine, The University of Hong Kong, Hong Kong SAR, China; ^5^ Modern Research Center for Traditional Chinese Medicine, Shanxi University, Taiyuan, China; ^6^ Guangdong Key Laboratory of Single Cell Technology and Application, Southern Medical University, Guangzhou, China

**Keywords:** matrine, metabolic network, Hepatocellular carcinoma, computational metabolomics, mechanism, MACS model

## Abstract

Hepatocellular carcinoma (HCC) is a complex issue in cancer treatment in the world at present. Matrine is the main active ingredient isolated from *Sophora flavescens* air and possesses excellent antitumor effects in HCC. However, the specific underlying mechanisms, especially the possible relationships between the anti-HCC effect of matrine and the related metabolic network of HCC, are not yet clear and need further clarification. In this study, an integrative metabolomic-based bioinformatics algorithm was designed to explore the underlying mechanism of matrine on HCC by regulating the metabolic network. Cell clone formation, invasion, and adhesion assay were utilized in HCC cells to evaluate the anti-HCC effect of matrine. A cell metabolomics approach based on LC-MS was used to obtain the differential metabolites and metabolic pathways regulated by matrine. The maximum activity contribution score model was developed and applied to calculate high contribution target genes of matrine, which could regulate a metabolic network based on the coexpression matrix of matrine-regulated metabolic genes and targets. Matrine significantly repressed the clone formation and invasion, enhanced cell–cell adhesion, and hampered cell matrix adhesion in SMMC-7721 cells. Metabolomics results suggested that matrine markedly regulated the abnormal metabolic network of HCC by regulating the level of choline, creatine, valine, spermidine, 4-oxoproline, D-(+)-maltose, L-(−)-methionine, L-phenylalanine, L-pyroglutamic acid, and pyridoxine, which are involved in D-glutamine and D-glutamate metabolism, glycine, serine and threonine metabolism, arginine and proline metabolism, etc. Our proposed metabolomic-based bioinformatics algorithm showed that the regulating metabolic networks of matrine exhibit anti-HCC effects through acting on MMP7, ABCC1, PTGS1, etc. At last, MMP7 and its related target *β*-catenin were validated. Together, the metabolomic-based bioinformatics algorithm reveals the effects of the regulating metabolic networks of matrine in treating HCC relying on the unique characteristics of the multitargets and multipathways of traditional Chinese medicine.

## Introduction

Hepatocellular carcinoma (HCC) is one of the diseases with high cancer-related deaths in the world (Yang and Heimbach, 2020). At present, the treatment of HCC is mainly based on surgery, combined with neoadjuvant therapy, adjuvant therapy, immunological therapy, and other comprehensive treatment methods in the clinical (Akateh et al., 2019). The encouraging achievements of molecular targeted therapy represented by sorafenib have enriched the treatment methods and provided us with new ideas for studying HCC (Tang et al., 2020). However, the initial incidence of HCC is hidden, and the early symptoms are not obvious (Njei et al., 2015). Most cases are already in the advanced stage when they are discovered, and miss the best treatment opportunity, which often leads to a poor prognosis (Ma et al., 2018). The main reason is that HCC is highly malignant and prone to metastasis and recurrence. Therefore, we still need to explore new anticancer strategies to improve the cure rate of HCC patients.

The abnormal change in cell metabolic network is an important feature of cancer (Martínez-Reyes and Chandel, 2021). With the disclosure of the Warburg effect, it has been found that tumor cells will change their metabolic pathways regulating the metabolic network to meet the unusual needs of proliferation, migration, and avoiding apoptosis (Martínez-Reyes and Chandel, 2021). Therefore, there is a close relationship between cancer and metabolic network. Metabolism is at the end of maintaining biological activity, which can reflect the influence of upstream levels on cancer cells. The metabolic network of organisms includes all biochemical reactions and related regulatory reactions (Biswas, 2015). Metabolic networks can analyze the intracellular response as a whole, avoiding the limitation caused by considering only one response module in organism, which is the advantage of a metabolic network (Frattaruolo et al., 2020). Therefore, regulation of an aberrant metabolic network has tremendous potential in the treatment of HCC.

In recent years, studies have shown that natural products have great potential in treating HCC. Matrine, extracted from the roots of *Sophora flavescens* air, is a type of alkaloid compound (Park et al., 2009). Studies have shown that it has excellent antitumor effects in many kinds of tumors and can significantly inhibit the proliferation of liver cancer cells (Ma et al., 2008), induce cell cycle arrest (Zhang et al., 2010), and induce autophagy (Zhang et al., 2010), etc., indicating its significant potential for cancer treatment and prognosis (Liu et al., 2014). However, the specific underlying mechanisms, especially the possible relationships between this anti-HCC effect of matrine and regulating metabolic network of HCC, are not yet clear and need further clarification.

Here, a maximum activity contribution score (MACS) was designed for the active contribution of genes to metabolic pathways, taking into account differential metabolites and core metabolic pathways, and quantified the contribution rate of genes to these core metabolic pathways. According to the results of the model, the core targets and underlying mechanisms of the regulating metabolism of matrine in treating HCC were decoded. Our study provided a methodological reference for the mechanism decode of matrine anti-HCC from the perspective of metabolic networks.

## Materials and Methods

### Reagents

The reagents in this study are described in Supplementary Material S1.

### Cell Culture and Treatment

The details of cell culture and treatment were performed as described in Supplementary Material S1.

### Cell Viability and Clone Formation Assay

Cell viability and clone formation assay were performed as described in Supplementary Material S1.

### Cell Invasion Assay

Referring to our previous method (Wang et al., 2021a), 1 and 2 mg/ml matrine were filled to the lower compartments for treating 24 h. The invading cells were observed under a microscope.

### Cell Adhesion Assay

Referring to our previous method with slight modification (Wang et al., 2021b), 1 and 2 mg/ml matrine were utilized to perform cell–cell adhesion and cell matrix adhesion assay.

### Sample Preparation for Metabolomics

Here, 6 × 10^4^ cells/mL of SMMC-7721 cells were inoculated in 90 × 20-mm dishes and were treated with 2 mg/ml matrine. Cell collection, extraction, and metabolomics study were carried out referring to our previous approach (Gao et al., 2020; Wang et al., 2021a; Wang et al., 2021b), and the detailed descriptions are shown in Supporting Information.

### Multivariate Data Analysis

UPLC-MS data files were obtained from Compound Discoverer 2.0 (Thermo Fisher, United States). Normalized data was imported into SIMCA-P software version 13.0 (Umetrics AB, Umea, Sweden) for gaining principal component analysis (PCA) and orthogonal partial least-squares discriminant analysis (OPLS-DA) for seeking differential metabolites (VIP > 1 and *p* < 0.05) between the control and matrine groups.

### Identification of Differential Metabolites

The differential metabolites were identified by referring to our previous approach (Wang et al., 2021a).

### Pathway Analysis

Metabolic pathways regulated by matrine were analyzed *via* metaboAnalyst, which is an enrichment analysis tool. Metscape (https://metscape.ncibi.org) was utilized to construct the metabolic network for obtaining metabolism-related genes regulated by matrine.

### Screening of Targets for Regulating Metabolism of Matrine

#### Targets Prediction of Matrine

Herbal Ingredients’ Targets Platform (Ye et al., 2011), similarity ensemble approach (Keiser et al., 2007), and Swiss Target Prediction (Daina et al., 2017) were applied to predict the targets of matrine.

### Construction of Coexpression Matrix of Matrine-Regulated Metabolic Genes and Targets

The expression data of matrine-regulated metabolic genes and targets were extracted from Microarray Datasets using the GEO (https://www.ncbi.nlm.nih.gov/geo) database, including GSE136247, GSE57957, and GSE36376 datasets. Sangerbox platform (https://www.sangerbox.com/) was used for gene analyses. The GSE136247 datasets contained 39 HCC tissue samples and 30 nontumor liver tissue samples, the GSE57957 datasets contained 39 HCC tissue samples and 39 nontumor liver tissue samples, and the GSE36376 datasets contained 240 HCC tissues samples and 193 nontumor liver tissue samples. The coexpression matrix was constructed by calculating the correlation between differential metabolite (NMR and LC-MS metabolomics)-related genes and targets.

### Maximum Activity Contribution Score Model Calculation

We defined the differential metabolites enrichment pathways with a *p* value of less than 0.05 as the core pathways, and a *p* value of greater than 0.05 as the noncore pathways. Mutual information between gene X and core pathway Y can be quantified as shown in the following equation:

$$I\left(X;Y\right)=\iint p\left(x,y\right)log\frac{p\left(x,y\right)}{p\left(x\right)p\left(y\right)}dxdy$$

where x and y represent realizations X and Y, I(X; Y) is the mutual information between the gene X and core pathway Y, p(x, y) is their joint probability mass function, and p(x) and p(y) are the marginal probability mass functions of X and Y, respectively. Mutual information between a set of input genes $\left\{x_{i}\in S_{m},i=1,\ldots \ldots ,m\right\}$ and an output core pathway Y can be estimated using the following formula:

$$I\left(S_{m};Y\right)=\iint p\left(S_{m},y\right).log\frac{p\left(S_{m},y\right)}{p\left(S_{m}\right).p\left(y\right)}dS_{m}dy=\int \ldots \ldots \overset{p\left(x_{1,}\ldots \ldots ,x_{m},y\right).log\frac{p\left(x_{1},\ldots \ldots ,x_{m},y\right)}{p\left(x_{1}\right)\ldots p\left(x_{m}\right).p\left(y\right)}}{\underset{dx_{1}\ldots dx_{m}dy}{\int }}$$

where $S_{m}$ is the set of m input genes. This formula explained that the applicability of mutual information reduced beyond the two-variable case even though it has robust measurement ability in a set of random variables.

To maximize the joint dependency of top-ranking genes on the contribution to core pathways, the redundancy among genes needs to be minimized, which requires incrementally selecting the maximally relevant genes while avoiding the redundant ones. In terms of mutual information, the purpose of causation factor selection is to find a factor set S with m genes $\;\left\{x_{i}\right\}$ , which have the highest mutual information value. Max relevance is to search satisfying factors, which approximates D (S, y) in mutual information calculation between genes and core pathways:

$$\max D\left(S,y\right),\;D=\frac{1}{\left|S\right|}\sum_{x_{i}\in S}I\left(x_{i};y\right)$$

Causation factors selected according to max relevance could likely have rich redundancy, i.e., the dependency among these factors could be large. When two factors depend highly on each other, the respective class discriminative power would not change much if one of them were removed. Therefore, the following minimal redundancy condition can be added to select mutually exclusive factors:

$$\min R\left(S\right),\;R=\frac{1}{{\left|S\right|}^{2}}\sum_{x_{i},x_{j}\in S}I\left(x_{i};x_{j}\right)$$

The ultimate aim is to find the genes set S with the MACS and directly optimize the following formula:

maxℵ(D,R),   ℵ=D−R

### Western Blot Assay

A total of 30 μg of protein was utilized to perform 10% SDS-PAGE for separation and transferred onto PVDF membranes. The primary antibodies MMP-7 (dilution 1:500), *β*-catenin (1:500), and GAPDH (1:1,000) were used in this analysis.

### Gene Ontology Enrichment and KEGG Pathways Analysis

The core targets of the regulating metabolism of matrine were used to perform GO enrichment and KEGG pathway analyses. GO analysis was performed by using the clusterProfiler (Yu et al., 2012) package of R software. KEGG database (Kanehisa and Goto, 2000) was used to perform KEGG pathway analyses.

### Construction of Matrine–Targets–Metabolic Genes–Metabolites–Metabolic Pathways Network

According to the calculation results of the MACS model, the core targets of matrine were selected to construct the multilevel network. The relationship between targets and metabolic genes was obtained from the String (https://string-db.org) database. Cytoscape (version 3.7.0) was utilized to integrate targets, metabolic genes, metabolites, and metabolic pathways and to establish a multilevel network.

### Statistical Analysis

Student’s *t*-test and one-way ANOVA were applied to calculate the significance of differences for two groups’ comparisons and multiple comparisons, respectively. The *p* value of <0.05 was considered to be statistically significant.

## Results

### Effects of Matrine on Cell Viabilities of L02 Cells and Invasion and Adhesion of SMMC-7721 Cells

**FIGURE 1:**
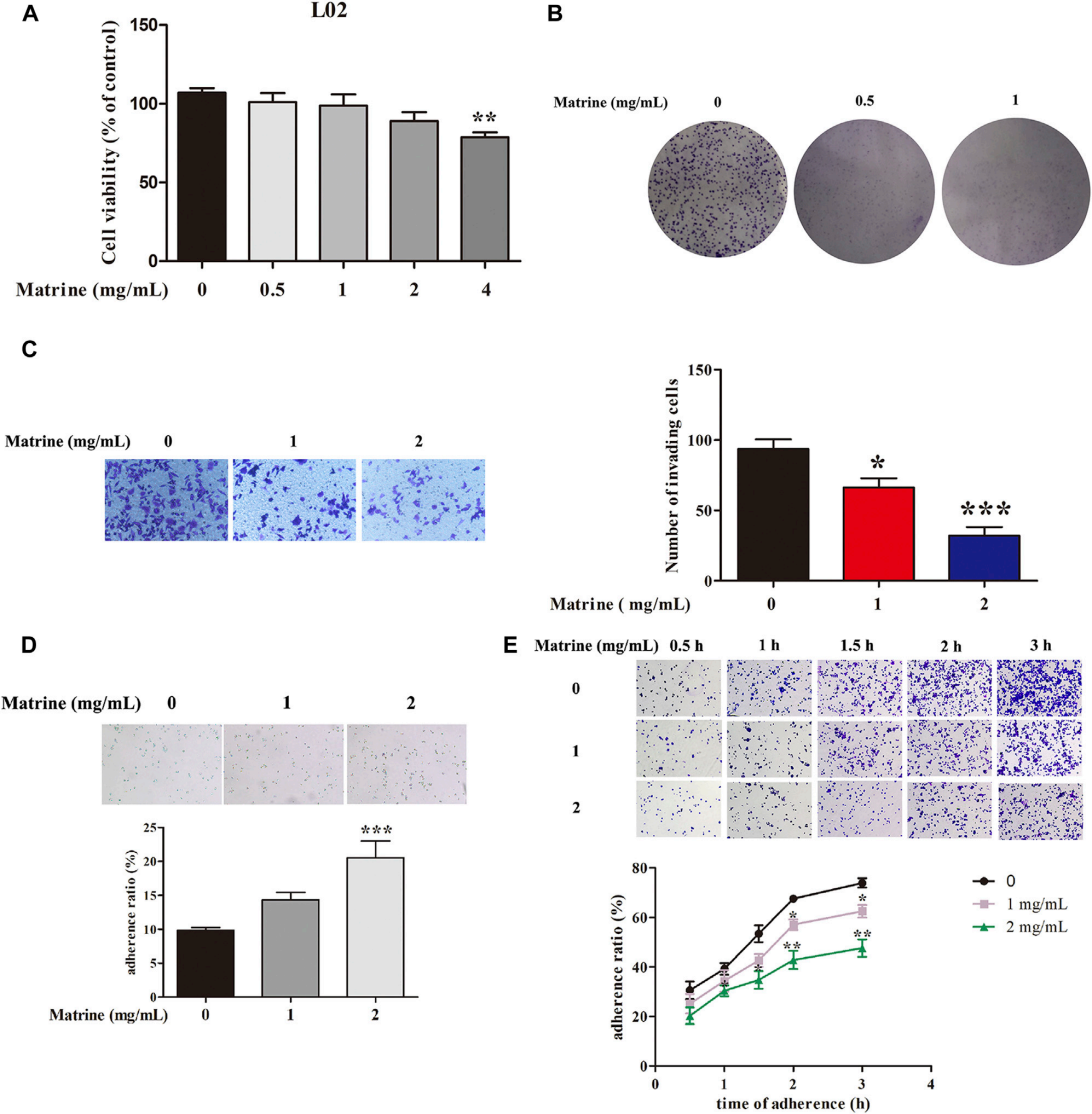
**(A)** Effects of matrine on the proliferation of L02 cells. **(B)** inhibitory effects of matrine on the clone formation, **(C)** invasion, **(D)** cell–cell adhesion, and **(E)** cell matrix adhesion in SMMC-7721 cells. Data are represented as mean ± SEM (*n* = 3). *p < 0.05, **p < 0.01, ***p < 0.001 as compared with control group.

The potential toxic effect of matrine on normal hepatic L02 cells was explored, and our results suggested that 0.5, 1, and 2 mg/ml matrine had almost no toxicity to L02 cells (Figure 1A). Our previous experiment has demonstrated that 0.5–4 mg/ml matrine prominently restrained the cell viability of SMMC-7721 cells at 24 h (Gao et al., 2018). Clone formation assay once again confirmed that 0.5 and 1 mg/ml matrine significantly inhibited the proliferation of SMMC-7721 cells at 48 h (Figure 1B). Our previous study showed that 1 and 2 mg/ml matrine could repress the migration of SMMC-7721 cells (Wang et al., 2017). Transwell cell invasion assay further observed the influence of matrine on the invasion ability of HCC cells. Compared with the control group, 1 and 2 mg/ml matrine can significantly inhibit the invasion of HCC cells, that is, the invasion ability of tumor cells gradually decreases with the increase of concentration (Figure 1C). For the adhesion assay, cell–cell adhesion results showed that 1 and 2 mg/ml matrine dramatically augmented the percentage of adhesion ratio of SMMC-7721 cells (Figure 1D), which suggested that matrine greatly enhanced the adhesion of SMMC-7721 cells to adjacent cells. In addition, 2 mg/ml matrine could markedly decrease the cell matrix adhesion at 1, 1.5, 2, and 3 h in SMMC-7721 cells (Figure 1E).

### Evaluation of the LC-MS Method

QC samples were used to evaluate the stability of the analytical system in the process of batch sample injection analysis. PCA analysis showed that QC samples were clustered together. We found that the quality control of the LC-MS method was stable, and the RSDs of the retention time and relative peak area were reliable for subsequent analysis.

### Matrine Regulates the Abnormality of Hepatocellular carcinoma Metabolic Profiles

**FIGURE 2:**
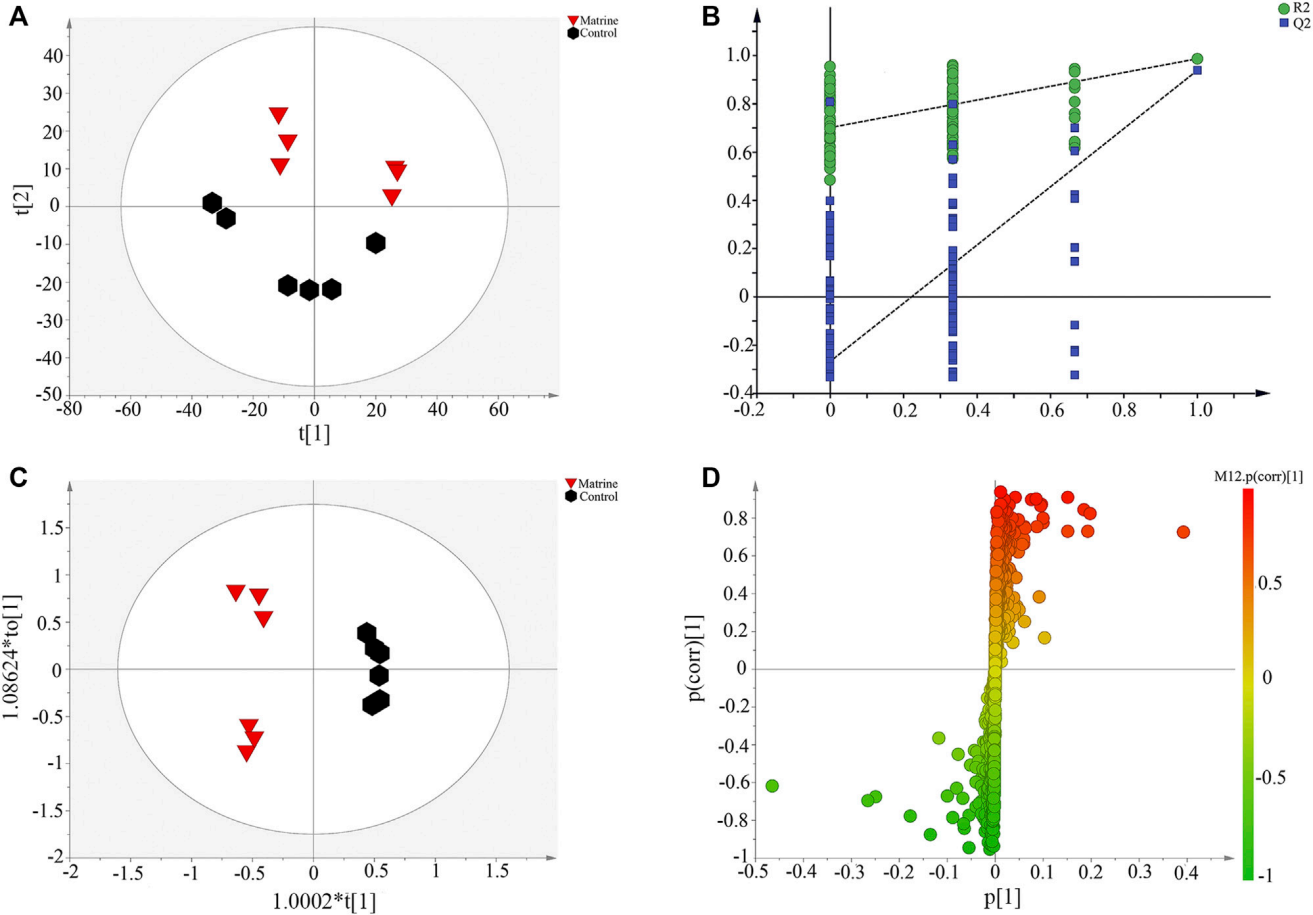
**(A**)Scatter plot of principal component analysis scores, **(B)** partial least-squares discriminant analysis model corresponding validation plot, **(C)** Scatter plot of orthogonal partial least-squares discriminant analysis (OPLS-DA) scores, and **(D)** S-plot based on OPLS-DA derived from SMMC-7721 cells.

To further explore the regulation of matrine on HCC metabolism, PCA was used to analyze the metabolic profile of all samples (Figure 2A). The control group and the matrine group were obviously separated along the t[1] axis, which further indicated that matrine had a significant effect on the metabolism of SMMC-7721 cells. The supervised pattern recognition method needs an external model verification method to arrange experiments to prove the validity of the model. Figure 2B shows that the slopes of the two regression lines are large, and the intercept between the lower regression line and the vertical axis is small, in which all *R* ^2^ and *Q* ^2^ values are lower than the original values, which indicates that the model verification is effective. Furthermore, OPLS-DA was applied to maximize the differences and to screen the vital metabolites that are responsible for the classification between the two groups (Figure 2C). S-plot (Figure 2D) was combined with VIP value to screen out metabolites with VIP > 1 and combined with *t*-test (*p* < 0.05) to get 10 differential metabolites and were identified through online databases (Supplementary Table S1).

**FIGURE 3:**
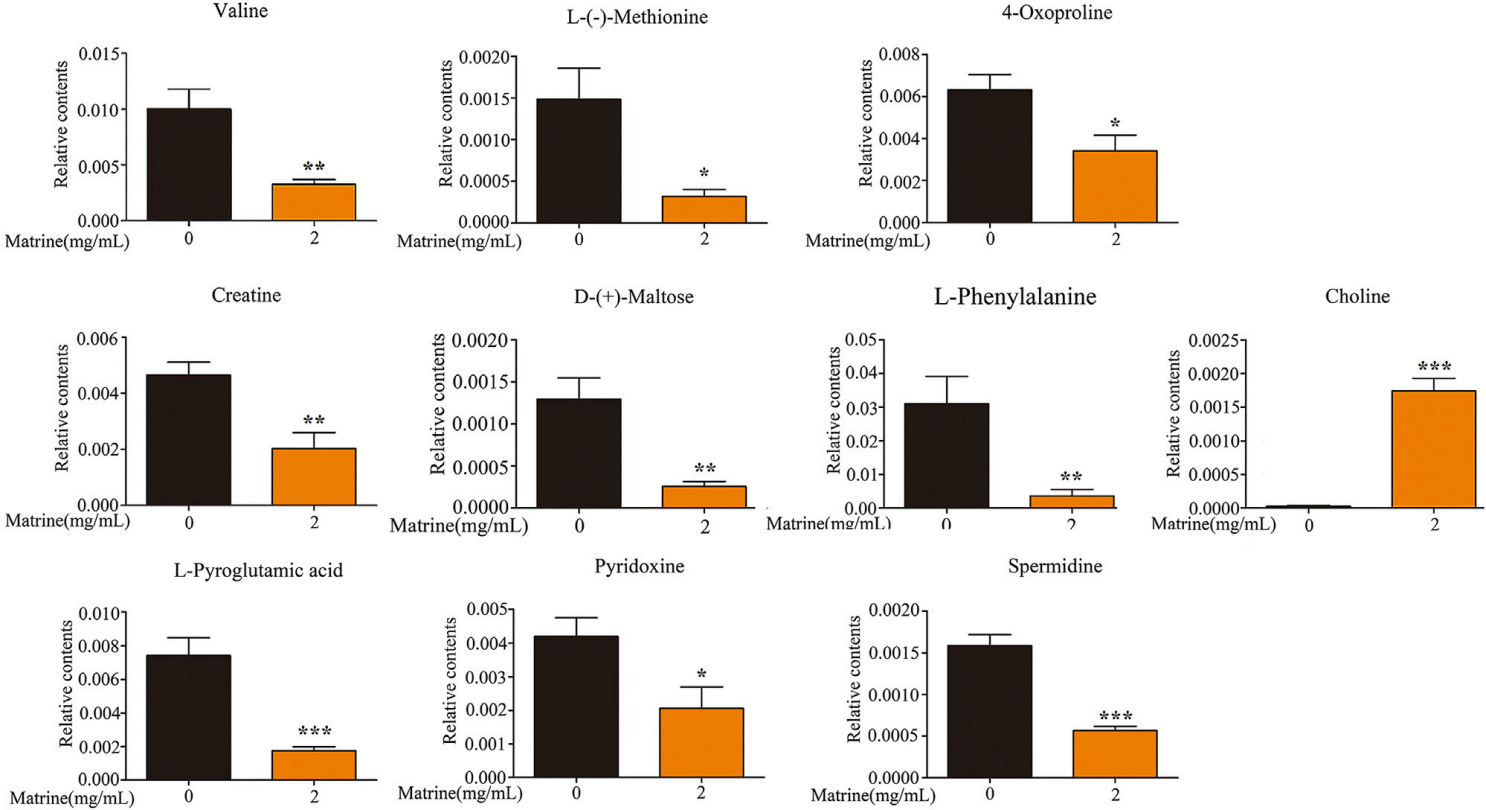
Levels of differential metabolites in SMMC-7721 cells after treatment of matrine. *p < 0.05, **p < 0.01, ***p < 0.001 versus control group.

**FIGURE 4:**
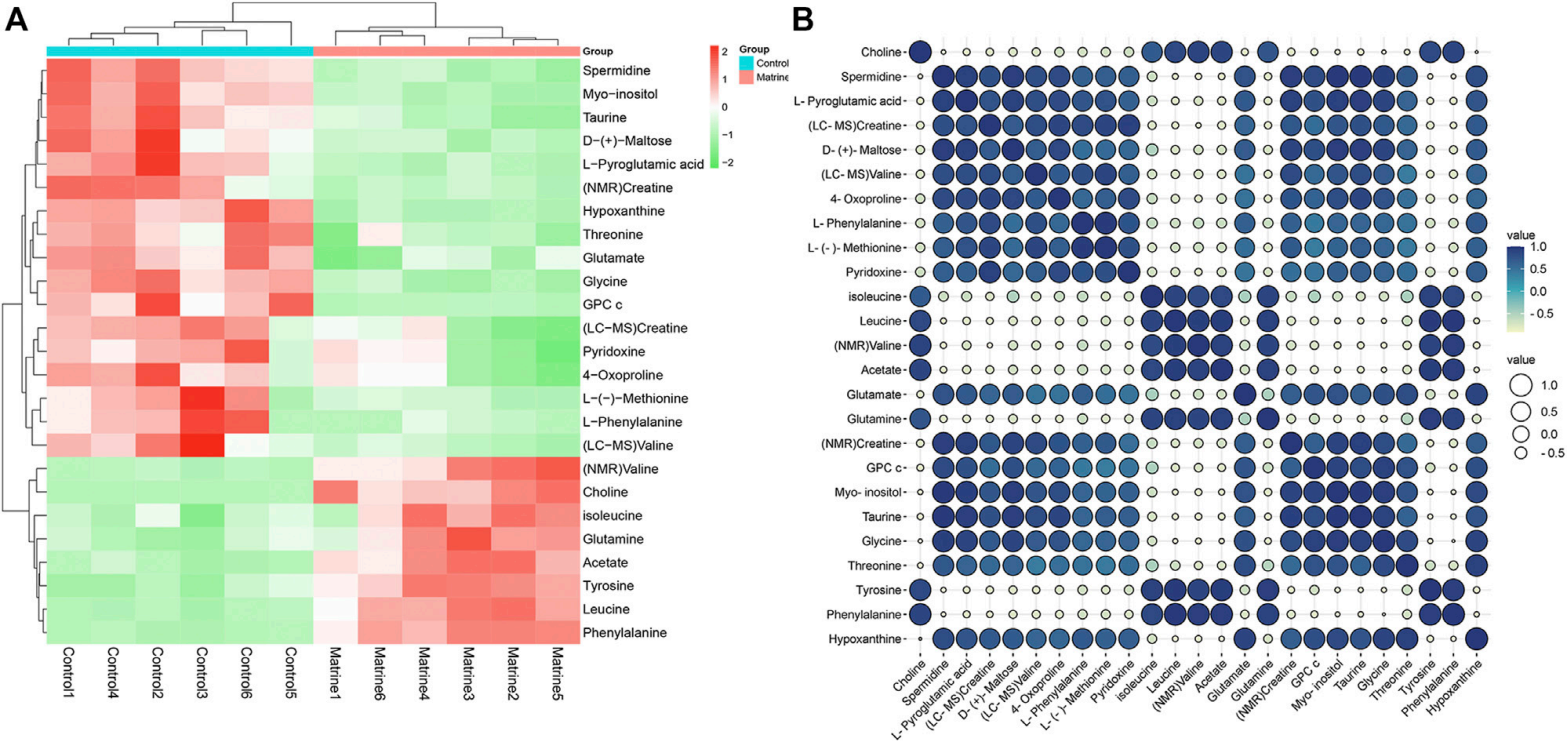
**(A)** Heat map and **(B)** correlation analysis of differential metabolites affected by matrine.

After matrine was administered, 10 differential metabolites were found in the cells. Compared with the blank group, after matrine intervention, the levels of L-(−)-methionine, 4-oxoproline, creatine, D-(+)-maltose, L-phenylalanine, L-pyroglutamic acid, pyridoxine, spermidine, and valine in cells decreased significantly, whereas the level of choline increased significantly (Figure 3). Heatmap analysis integrating LC-MS and previous NMR metabonomics results showed that the metabolic profile of the matrine group obviously deviated from the control group (Figure 4A).

### Correlation Analysis

Pearson correlation analysis was utilized to expound the correlations between differential metabolites. The results indicated that there exist positive correlations among isoleucine, leucine, valine, and acetate and that they have strong negative correlations with creatine, pyridoxine, taurine, hypoxanthine, and glycine. In addition, choline has strong negative correlations with valine, glycine, and hypoxanthine (Figure 4B). These results show that different metabolites are interrelated with each other.

### Metabolic Pathways Analysis

**FIGURE 5:**
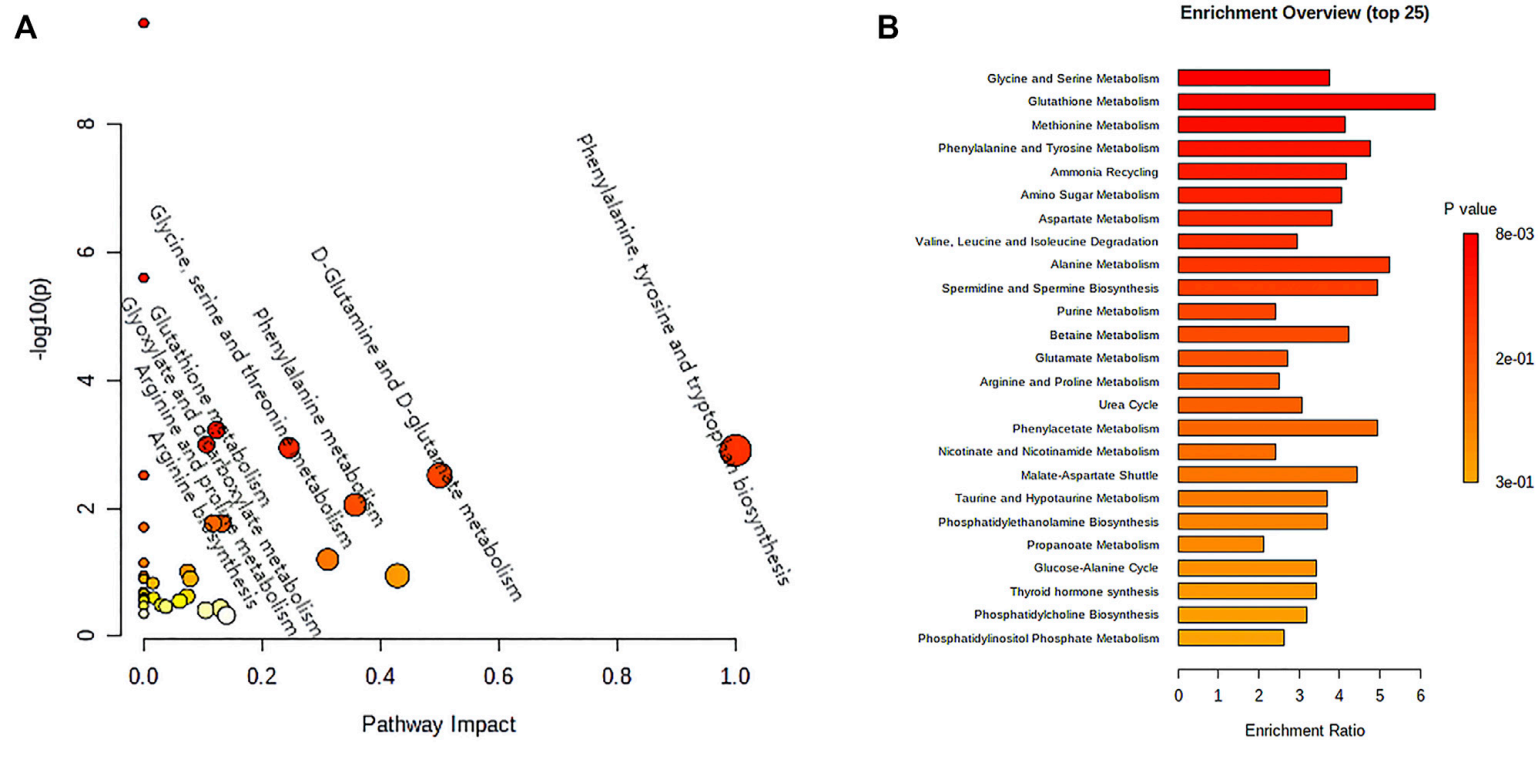
MetPA analysis of **(A)** the metabolic pathway and **(B)** the enrichment of metabolite sets.

All differential metabolites from LC-MS and NMR metabolomics are input into metaboanalyst for pathway enrichment analysis, and the results of MetPA pathway analysis are shown in Figure 5A. Combining Holm *p* value, FDR, and Impact value, it was found that matrine could significantly interfere with eight metabolic pathways, including phenylalanine, tyrosine and tryptophan biosynthesis, d-glutamine and d-glutamate metabolism, phenylalanine metabolism, glycine, serine, and threonine metabolism, Arginine and proline metabolism, glutathione metabolism, arginine biosynthesis, and glyoxylate and dicarboxylate metabolism. The results of metabolite set enrichment analysis (Figure 5B) further supported the MetPA pathway.

### Metabolism-Related Genes Regulated by Matrine

**FIGURE 6:**
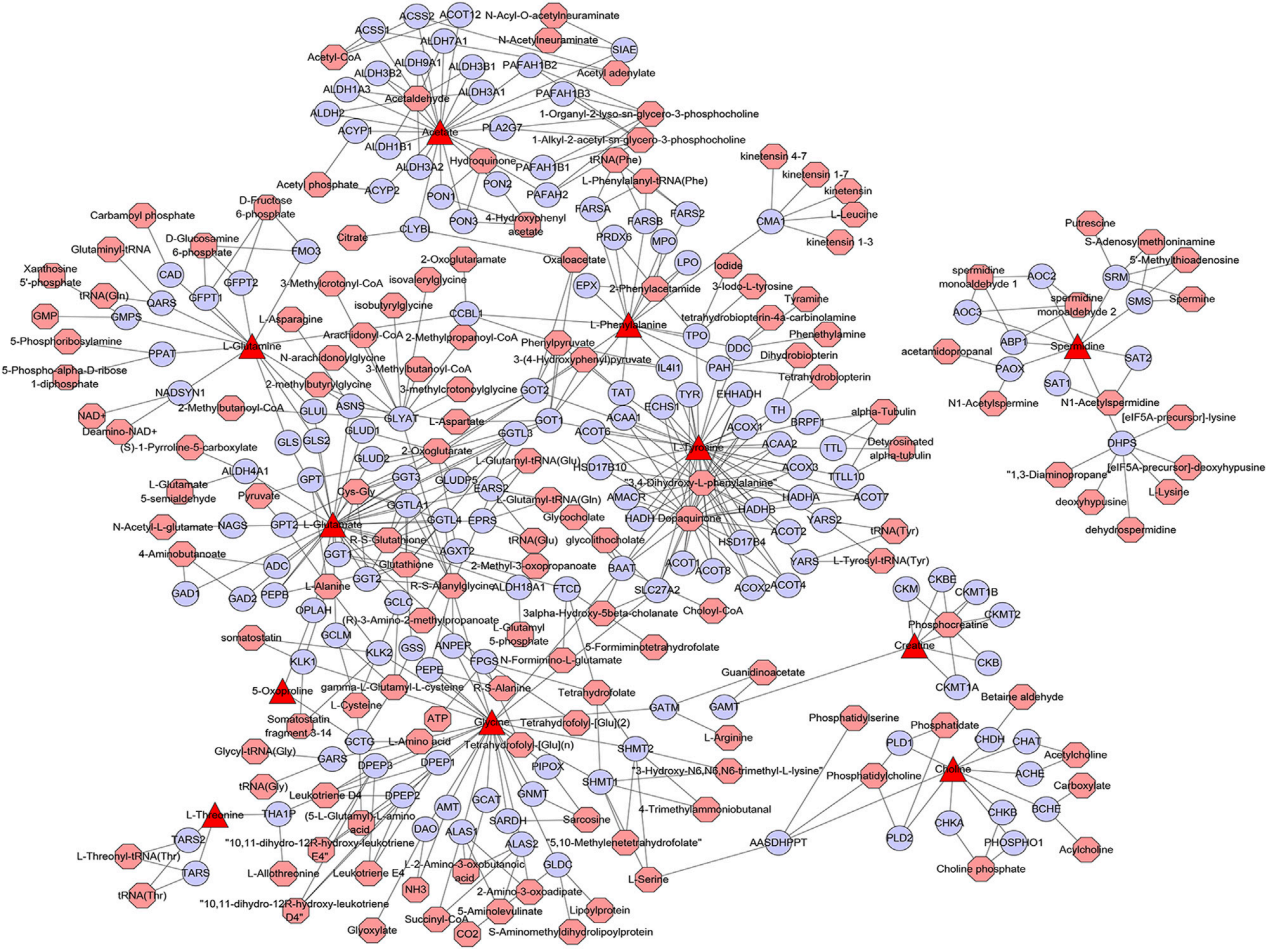
Network of differential metabolites-genes of matrine for its antihepatocellular carcinoma effect. The figure was constructed using metscape: the triangle nodes represent differential metabolites, the circular nodes represent metabolic genes, and the octagon nodes represent differential metabolite-related metabolites.

A metabolic network with metabolites and metabolic enzymes as the core is the material and energy basis of cell life activities, and the canceration process of tumor cells is frequently accompanied by metabolic remodeling at the molecular level (Noda-Garcia et al., 2018). For example, the mutation of the metabolic enzyme gene or the change of expression level will lead to significant changes in the intracellular content of metabolites, and this metabolic rearrangement phenomenon can play a key role in tumor evolution (Morandi and Indraccolo, 2017). To search the core genes associated with differential metabolites and metabolic pathways affected by matrine, the differential metabolites in eight metabolic pathways were utilized to construct a metabolite–gene network. There were 158 metabolism-related genes regulated by matrine, including ALDH2, ALAS1, CKB, GGT1, AGXT2, and PRDX6 (Figure 6). Seo et al. (2019) showed that ALDH2 deficiency exacerbates alcohol-associated HCC development both in patients and mouse models. It has been reported that the expression of AGXT2 was downregulated in HCC and correlated with a poor prognosis for HCC patients (Cai et al., 2020). It can be seen that these metabolism-related genes are closely related to the occurrence and development of HCC.

### Targets for Regulating Metabolism of Matrine Extracted Based Maximum Activity Contribution Score Model

**FIGURE 7:**
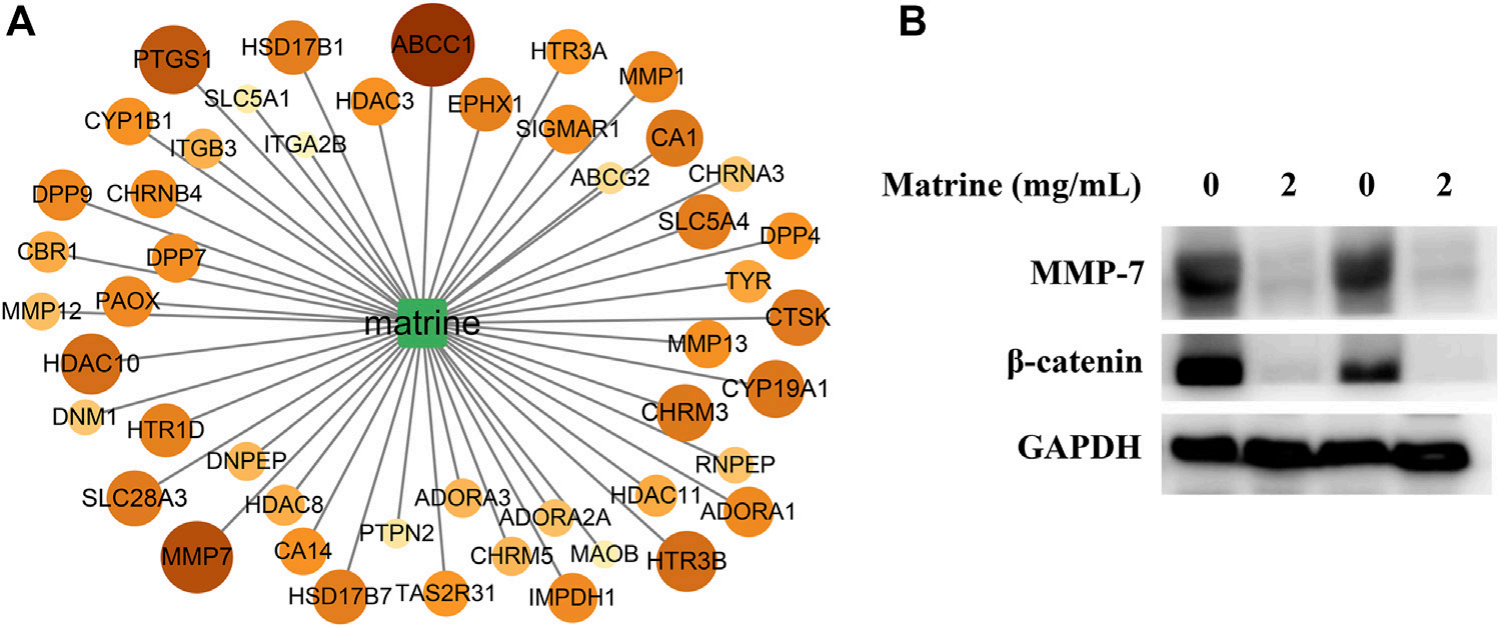
**(A)** Value distribution of maximum activity contribution score mode of core targets in matrine, and the darker the color, the bigger the value. **(B)** effect of matrine on the protein levels of MMP-7 and *β*-catenin in SMMC-7721 cells.

To seek the core targets for regulating the metabolism of matrine, we designed a scoring model of the gene’s active contribution to metabolic pathways, taking into account the differential metabolites and core metabolic pathways. The score of each target of matrine was calculated (Figure 7A). According to the calculation results, 48 targets with scores higher than the average were reserved as core targets of matrine to regulate metabolism and exert anti-HCC, including MMP7, ABCC1, PTGS1, HTR3B, HDAC10, and CYP19A1. MMP7 is also called matrilysin, which plays the role of a metastatic factor by promoting the migration and invasion of cancer cells; overexpression of MMP7 was found in HCC (Scheau et al., 2019). MMP7 is an important target downstream of the *β*-catenin signaling pathway (Chen et al., 2013). Therefore, MMP7 and its upstream target *β*-catenin were chosen to further elucidate the potential mechanism of matrine anti-HCC. As shown in Figure 7B, matrine significantly inhibited the expression of MMP7 and *β*-catenin in SMMC-7721 cells.

### KEGG Pathways and Gene Ontology Enrichment Analysis

**FIGURE 8:**
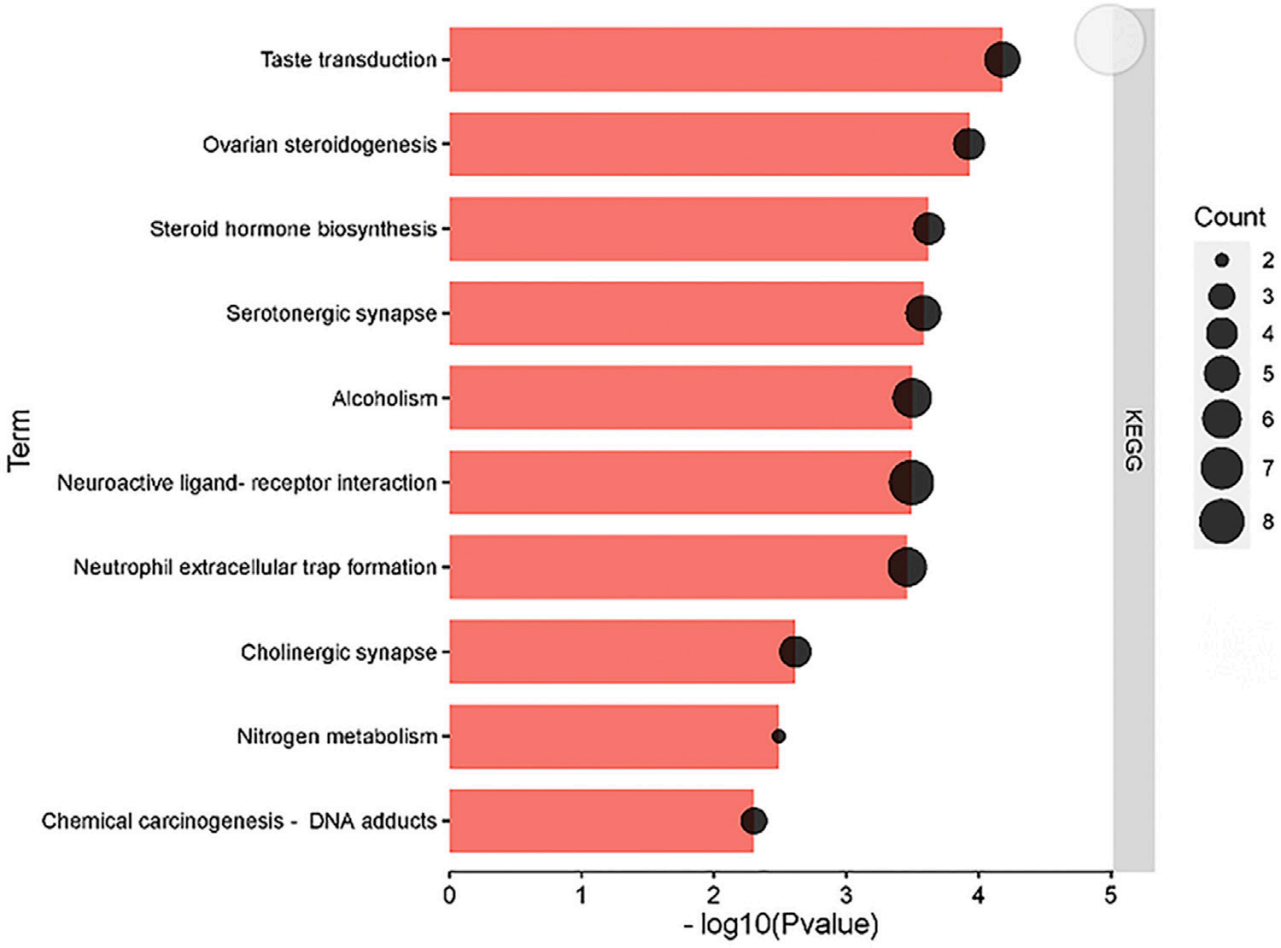
KEGG pathway analysis of the core targets of the regulating metabolic networks of matrine.

To further interpret the underlying mechanism of the regulating metabolism of matrine in treating HCC, KEGG pathways were enriched by core targets of the regulating metabolism of matrine (Figure 8). Our results suggested that matrine treating HCC is mainly through the following pathways: Neuroactive ligand–receptor interaction, nitrogen metabolism, cholinergic synapse, serotonergic synapse, etc.

**FIGURE 9:**
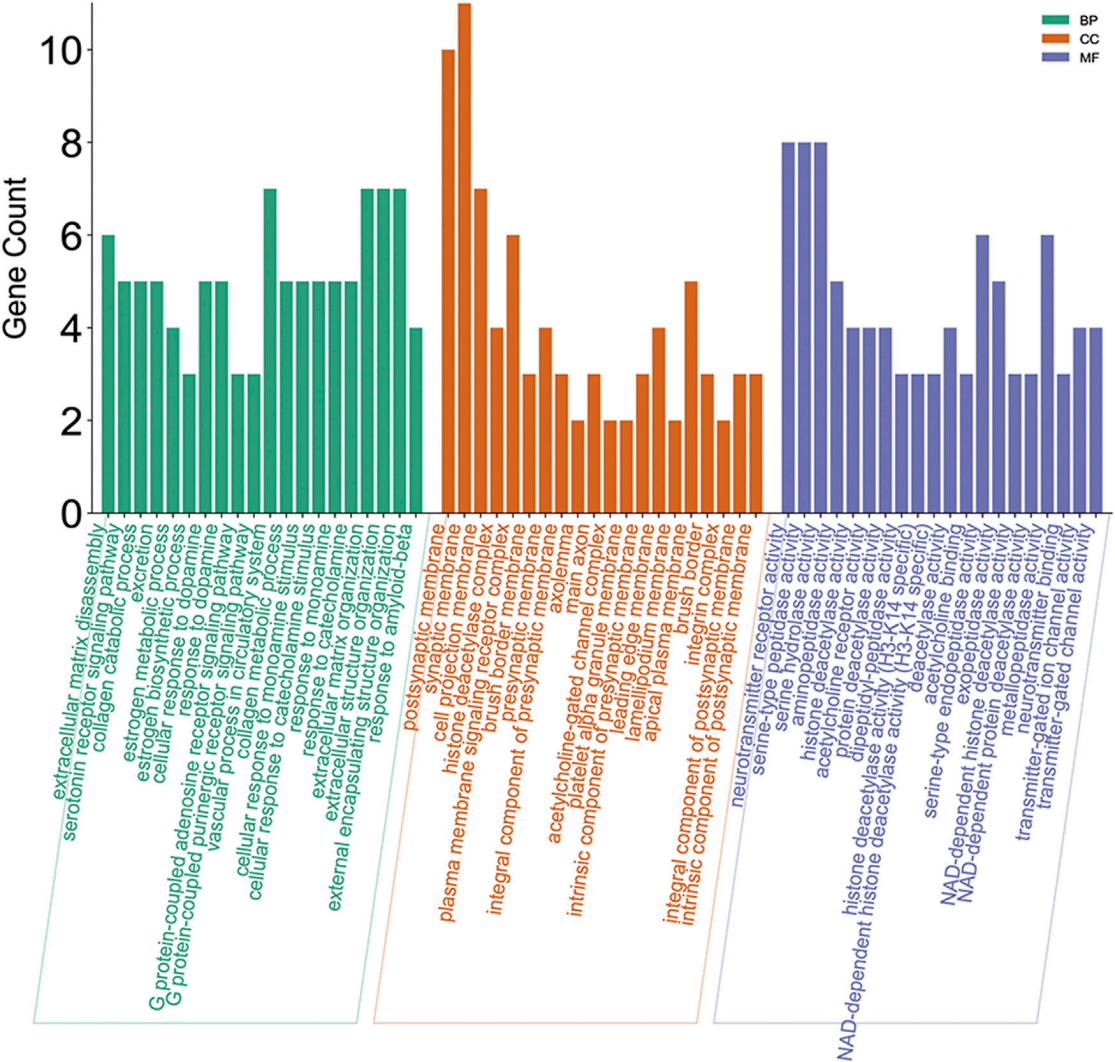
Gene ontology enrichment processing of the core targets of the regulating metabolic networks of matrine.

The results of GO enrichment containing biological processes (BP), molecular functions (MF), and cellular components (CC) are shown in Figure 9. MF results showed that the regulating metabolism of matrine treating HCC is through neurotransmitter receptor activity, serine-type peptidase activity, etc. Matrine regulates the GO BP of HCC including extracellular matrix disassembly and estrogen metabolic process.

### Multilevel Network of Matrine Regulating Metabolism and Its Targets

**FIGURE 10:**
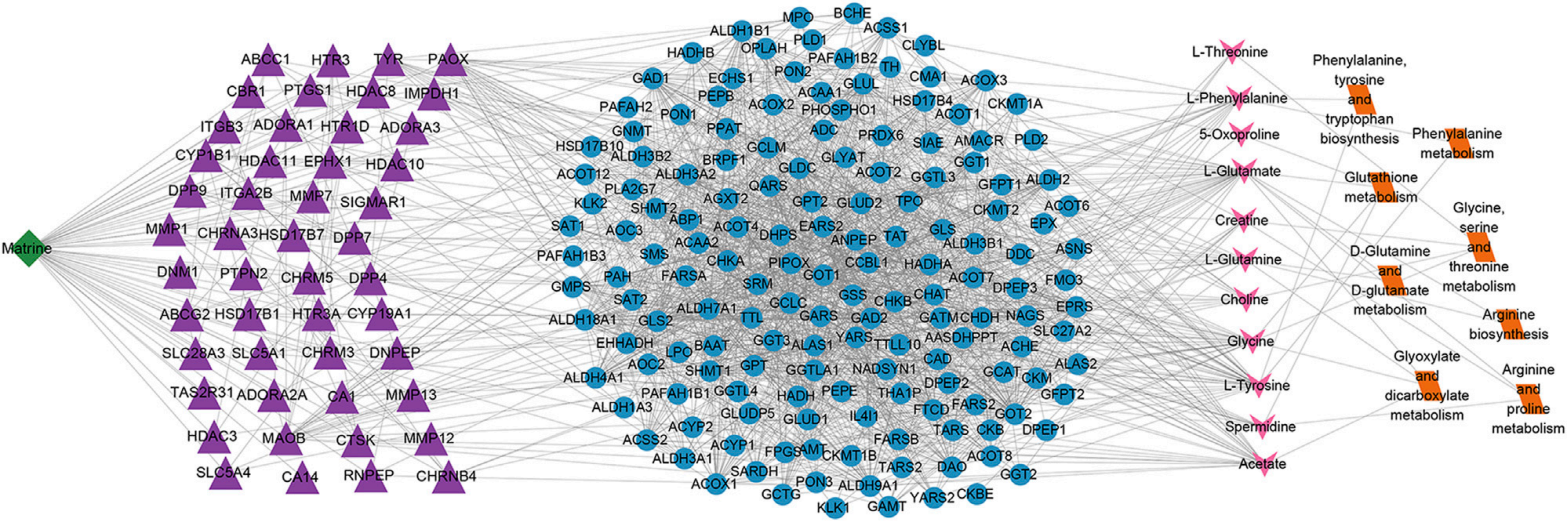
Matrine–targets–metabolic genes–metabolites–metabolic pathways network. The nodes of matrine, vital targets, metabolic genes, metabolites, and metabolic pathways were presented in green, purple, blue, pink, and orange, respectively.

To further clarify the metabolic mechanism of matrine against HCC, a multilevel network of matrine was established (Figure 10). The results showed that matrine possibly acts on 48 vital targets (MMP7, ABCC1, PTGS1, HTR3B, HDAC10, etc.); thus, affecting 158 metabolism-related genes, finally resulting in the alteration of 11 metabolites and 8 metabolic pathways.

**FIGURE 11:**
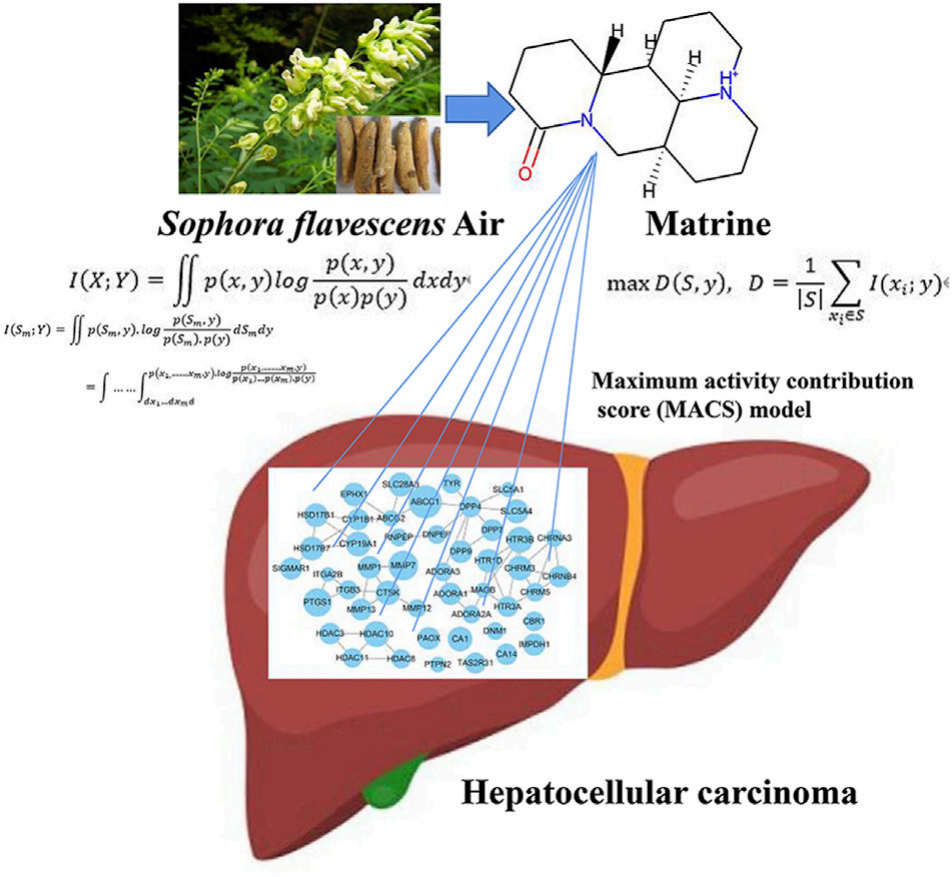
The schematic diagram of matrine mediated metabolic network anti-HCC based on computational metabolomics approach.

## Discussion

HCC itself is a major health burden and the second leading cause of cancer-related death (Kim et al., 2019). Sorafenib is the only new targeted drug for HCC approved by FDA (Dutta and Mahato, 2017). Therefore, it is urgent to find more effective drugs to treat HCC. In recent years, great progress has been made in anti-HCC research in traditional Chinese medicine (TCM). TCM has unique advantages in inhibiting tumor cell proliferation, stabilizing tumor growth, improving symptoms and signs, reducing toxicity and increasing efficiency, preventing recurrence, improving quality of life, and prolonging survival time (Liu et al., 2019). Numerous studies have certificated that matrine can exert strong anti-HCC activities. Our previous study also showed that matrine significantly inhibited the migration of HCC cells (Wang et al., 2017). However, the potential mechanisms of matrine against HCC were still unclear.

Compared with normal cell metabolism, the metabolic network of cancer cells will be reprogrammed to meet the needs of bioenergy and biosynthesis (Pavlova and Thompson, 2016). Metabolic reprogramming is a phenomenon that tumor cells rapidly proliferate by changing the energy metabolism mode to quickly adapt to the microenvironment such as hypoxia, acidity, and nutrient deficiency (Boroughs and DeBerardinis, 2015). Therefore, a metabolic network plays a key role in the pathogenesis of HCC. TCM often regulates the abnormal metabolic network of HCC to play a therapeutic role, but how this regulation is driven is still unclear.

In this study, a new bioinformatics algorithm was proposed to decode the underlying mechanism of the regulating metabolic networks of matrine against HCC. MACS model was used to calculate the core targets of the regulating metabolic network of matrine in treating HCC by taking into account both differential metabolites and core metabolic pathways. The results will provide new strategies and methodological references for the research of other TCM in treating tumors by regulating metabolic networks.

During the development of cancer, many metabolic pathways are involved in abnormal metabolic network regulation. Creatine is involved in glycine, serine, and threonine metabolism and arginine and proline metabolism. A study showed that creatine is one of the energy sources during the growth of tumor cells (Kazak and Cohen, 2020). After matrine intervention, the creatine level decreased significantly, indicating that matrine may inhibit the proliferation of HCC cells by regulating the creatine level. Choline is involved in glycine, serine, and threonine metabolism. Choline can improve the fatty acid utilization rate and prevent abnormal accumulation of liver fat (Huang et al., 2012). The lipid metabolism of HCC is abnormal due to the insufficient ability of lipid catabolism and anabolism. Abnormal choline metabolism is a common feature of cancer. Zhang et al. (2017) found that choline was closely related to the pathological features of esophageal cancer such as lymph node metastasis based on metabolomics. Matrine can significantly increase the amount of intracellular choline, indicating that matrine can improve the lipid metabolism disorder of HCC. In summary, matrine may inhibit the proliferation of HCC cells by regulating choline and creatine levels.

We used the MACS algorithm to infer the core targets of the regulating metabolic network of matrine. It was found that ABCC1, PTGS1, MMP7, etc. have higher scores. ABCC1 is a member of the ABC transporter family subfamily. In general, ABCC1 can excrete harmful substances from the body; thus, protecting the tissues. Clinical studies show that ABCC1 is upregulated in nonsmall cell lung cancer, breast cancer, ovarian cancer, renal cancer, and liver cancer, which is positively correlated with clinical–pathological stage and metastasis (Cao et al., 2017; Yamada et al., 2018). PTGS-induced eicosanoid metabolism has been reported to be related to many types of cancers (Wang and Dubois, 2010). PTGS1 is one of the common types of PTGSs. Recent evidence that supports the suppression of PTGS1 was also demonstrated to exhibit anticancer properties, including HCC (Gupta et al., 2003; Cusimano et al., 2007; Cho et al., 2013). MMP-7 is a member of the family of zinc-dependent endoproteases to degrade the extracellular matrix, which could participate in various processes in malignancies, including cell proliferation and migration and cell apoptosis (Cui et al., 2017). This further demonstrated that the core targets screened for regulating metabolic networks were reliable in treating HCC.

In addition, the matrine–targets–metabolic genes–metabolites–metabolic pathways network was constructed to decode the metabolic mechanism of matrine against HCC. In the network, it was predicted that matrine exerted anti-HCC effect has the characteristics of multilinks and multilevels possibly through acting on the vital targets (MMP7, ABCC1, PTGS1, HTR3B, HDAC10, etc.); thus, affecting 158 metabolism-related genes (FPGS, SIAE, GCLM, GCLC, HADHA, MPO, etc.), eventually affecting metabolic pathways.

## Conclusion

Overall, a metabolomics-based bioinformatics algorithm was proposed to illustrate that core targets and underlying mechanisms of the regulating metabolic networks of matrine exert the therapeutic role of HCC. This study provided new strategies and methodological references for interpreting the underlying mechanisms of TCM in treating HCC based on metabolic networks.

## Data Availability

The original contributions presented in the study are included in the article/Supplementary Material; further inquiries can be directed to the corresponding authors.

## Abbreviations

HCC, Hepatocellular carcinoma; MACS, Maximum activity contribution score; OPLS-DA, Orthogonal partial least-squares discriminant analysis; PCA, Principal component analysis; BP, Biological processes; CC, Cellular components; GO, Gene Ontology; MF, Molecular functions; TCM, Traditional Chinese medicine.
