# Supplementary material for: Computational Metabolomics Reveals the Potential Mechanism of Matrine Mediated Metabolic Network Against Hepatocellular Carcinoma
Source: Front Cell Dev Biol. 2022 Jul 22;10:859236. doi: 10.3389/fcell.2022.859236 (PMC9354776; doi:10.3389/fcell.2022.859236)
Supplement: Supplementary file 1 [file Table1.docx]

Table S1. Identified differential metabolites in the SMMC-7721 cells.

| **No.** | **Metabolies** | **m/z** | **T_R_(min)** | **ion** |
| --- | --- | --- | --- | --- |
| 1 | 4-Oxoproline | 129.04179 | 3.284 | M+H |
| 2 | D-(+)-Maltose | 364.09815 | 1.912 | M-H |
| 3 | L-(-)-Methionine | 151.02452 | 3.092 | M+H |
| 4 | L-Phenylalanine | 165.07895 | 7.043 | M-H |
| 5 | L-Pyroglutamic acid | 129.04271 | 3.271 | M-H |
| 6 | Pyridoxine | 169.07387 | 3.581 | M+H |
| 7 | Choline | 99.99622 | 2.277 | M+H |
| 8 | Creatine | 131.06954 | 1.83 | M-H |
| 9 | Valine | 117.07918 | 2.384 | M+H |
| 10 | Spermidine | 145.15796 | 1.436 | M-H |
