# Supplementary material for: Computational Metabolomics Reveals the Potential Mechanism of Matrine Mediated Metabolic Network Against Hepatocellular Carcinoma
Source: Front Cell Dev Biol. 2022 Jul 22;10:859236. doi: 10.3389/fcell.2022.859236 (PMC9354776; doi:10.3389/fcell.2022.859236)
Supplement: Supplementary file 2 [file DataSheet1.docx]

**Supplemental Data:**

**Supplemental Experimental Procedures:**

**Reagents**

Matrine (≧98 % purity by HPLC) was obtained from Jingzhu Biotechnology Co., Ltd. (Nanjing, China). High glucose Dulbecco’s modified Eagle medium (DMEM; 4.5 g/L), fetal bovine serum (FBS), 0.25% trypsin, and 3-(4,5-dimethylthiazol-2-yl)-2,5-diphenyltetrazolium bromide (MTT) were purchased from Shanghai Sangon Biotechnology Co. Ltd. (Shanghai, China). Transwell invasion chamber was purchased from corning costar company (Andover, MA, USA). Matrigel was purchased from BD (Biosciences, Bedford, MA, USA).

**Cell culture and treatment**

Human normal hepatic L02 cells and human hepatoma SMMC-7721 cells were purchased from Nanjing EnoGene Biotechnology (Nanjing, China) and Boster Biological Technology co., Ltd (Wuhan, China), respectively. Cells were cultured in incubator at 37℃ with DMEM medium containing 10% FBS. When the cells reached 80% confluency, they were exposed to different concentrations of matrine (0.5, 1, 2 and 4 mg/mL) for 24 h.

**Cell viability assay**

L02 cells (1 × 10^4^ cells/well) were seeded in 96-well plates, and treated with different concentrations of matrine (0, 0.5, 1, 2, and 4 mg/mL). MTT was superinduced to 96-well plate for 4 h, then the culture supernatant was removed. Finally, DMSO was utilized to dissolve the purple crystals. The plate reader was utilized to detect the absorbance at 570 nm.

**Clone formation assay**

SMMC-7721 cells (300 cells/well) were seeded in 6-well plates. After treatment with matrine (0.5 and 1 mg/mL) for 48h, the cells were cultured for 14 days. The supernatant was displaced every 3 days. In the end, the crystal violet solution was utilized to fix and stain cells, then the cells were photographed under a microscope to judge colony formation.

**Metabolomics study**

**The detailed descriptions of sample extraction and UPLC-MS/MS methods.**

**1 Sample extraction**

The steps for collecting cell samples were as follows: The cells were washed three times with PBS, 2 ml of 80 % (v/v) methanol (cooled to -80 °C) was added to plates and incubated for 20 min at -80 °C. The cells were scraped and transferred to a 10-ml tube on ice and sonicated for 10 min. After incubation for 1h at -20 ℃, the methanol mixture was centrifuged at 14,000 rpm for 5 min at 4 °C. The above ultrasonic sieving was repeated and the supernatant was collected two times in 5-mL EP tubes for lyophilization.

**2 LC-MS methods**

For LC-MS preparation, the lyophilized cell extract was reconstituted with 60 μl of 0.1% formic acid solution. After centrifugation at 13,000 rpm, 4 °C for 10 min, the supernatant was used for subsequent analysis. LC–MS data were acquired using Dionex UltiMate 3000 UHPLC-Q Exactive Orbitrap-MS and Xcalibur workstation (Thermo Fisher Scientific Inc., Waltham, Ma, USA). Mobile phases A and B were composed of 0.1% aqueous formic acid and acetonitrile, respectively, at a flow rate of 0.2 mL/min. Sample analysis was carried out under positive and negative ion modes, using heated electrospray ionization (HESI). The scan mode was Full Scan/dd-MS2. The spray voltage was 3.5 kV for the positive mode and 2.5 kV for the negative mode, the capillary temperature was 320 °C and the sheath and auxiliary gas flow rates were 35 and 10 arbitrary units, respectively. The mass scanning range was m/z 100–1500. Chromatographic separation was performed on an Acquity UPLC HSS T3 column. Elution by time gradient: 0-2 min, 1%-1% B; 2-8 min, 1%-35% B; 8-11 min, 35%-40% B; 11-14 min, 40%- 60% B; 14-17 min, 60%-99% B; 17-18 min, 99%-99% B; 18-19 min, 99%-1% B; 19-20 min, 1%-1% B. The injection volume was 10 μL. For every eight samples, a QC sample was inserted to monitor changes throughout the analysis.
